# A dynamic model for estimating adult female mortality from ovarian dissection data for the tsetse fly *Glossina pallidipes* Austen sampled in Zimbabwe

**DOI:** 10.1371/journal.pntd.0005813

**Published:** 2017-08-30

**Authors:** Sarah F. Ackley, John W. Hargrove

**Affiliations:** 1 Department of Epidemiology and Biostatistics, University of California San Francisco, San Francisco, California, United States of America; 2 Proctor Foundation, University of California San Francisco, San Francisco, California, United States of America; 3 Centre of Excellence in Epidemiological Modelling and Analysis (SACEMA), University of Stellenbosch, Stellenbosch, South Africa; National Institute of Allergy and Infectious Diseases, UNITED STATES

## Abstract

Human and animal trypanosomiasis, spread by tsetse flies (*Glossina* spp), is a major public health concern in much of sub-Saharan Africa. The basic reproduction number of vector-borne diseases, such as trypanosomiasis, is a function of vector mortality rate. Robust methods for estimating tsetse mortality are thus of interest for understanding population and disease dynamics and for optimal control. Existing methods for estimating mortality in adult tsetse, from ovarian dissection data, often use invalid assumptions of the existence of a stable age distribution, and age-invariant mortality and capture probability. We develop a dynamic model to estimate tsetse mortality from ovarian dissection data in populations where the age distribution is not necessarily stable. The models correspond to several hypotheses about how temperature affects mortality: no temperature dependence (model 1), identical temperature dependence for mature adults and immature stages, *i*.*e*., pupae and newly emerged adults (model 2), and differential temperature dependence for mature adults and immature stages (model 3). We fit our models to ovarian dissection data for *G*. *pallidipes* collected at Rekomitjie Research Station in the Zambezi Valley in Zimbabwe. We compare model fits to determine the most probable model, given the data, by calculating the Akaike Information Criterion (AIC) for each model. The model that allows for a differential dependence of temperature on mortality for immature stages and mature adults (model 3) performs significantly better than models 1 and 2. All models produce mortality estimates, for mature adults, of approximately 3% per day for mean daily temperatures below 25°C, consistent with those of mark-recapture studies performed in other settings. For temperatures greater than 25°C, mortality among immature classes of tsetse increases substantially, whereas mortality remains roughly constant for mature adults. As a sensitivity analysis, model 3 was simultaneously fit to both the ovarian dissection and trap data; while this fit also produces comparable mortality at temperatures below 25°C, it is not possible to obtain good fits to both data sources simultaneously, highlighting the uncertain correspondence between trap catches and population levels and/or the need for further improvements to our model. The modelling approach employed here could be applied to any substantial time series of age distribution data.

## Author summary

Trypanosomiasis, spread by tsetse flies (*Glossina* spp.), is a disease that is fatal for both humans and livestock if left untreated, and is a serious threat to public health in many regions of sub-Saharan Africa. In order to understand the dynamics of the disease it is important also to understand tsetse population dynamics. Tsetse fly mortality estimates are central to this understanding, but are difficult to acquire from wild populations. Previous methods for estimating mortality from age-distribution data assume a stable age structure and age-invariant mortality and capture probability. Based on prior fieldwork, none of these assumptions appears justified. Building on previous mortality estimation techniques, and incorporating what is known about tsetse population dynamics, we develop simulation techniques to estimate mortality for tsetse populations where the age distribution is not necessarily stable. We fit our models to age-distribution data produced in 1991 and 1992 at Rekomitjie Research Station in the Zambezi Valley in Zimbabwe. Our final model produces mortality estimates consistent with those of mark-recapture studies performed in other settings. We find that mortality increases with temperature, a result consistent with field and laboratory findings, and that the temperature effects are much more severe for pupae and newly emerged adults than for mature adults. Our dynamical modelling approach could be used for mortality estimation for any population where substantial age distribution data are available: specifically, it could be used to answer substantive questions about tsetse flies in other settings.

## Introduction

Human and animal trypanosomiasis, which is spread by tsetse flies (*Glossina* spp), is a major health concern in much of sub-Saharan Africa [1–3]. Research on trypanosomiasis and tsetse has been carried out for nearly 60 years at Rekomitjie Research Station in the Zambezi Valley (16° 18' S, 29° 23' E; altitude 500 m) [4]. Studies at the station have provided improved understanding of vector and disease dynamics, with the aim of improving disease control [5–7]. It has been shown that the basic reproduction number of vector borne diseases such as trypanosomiasis is strongly dependent on vector mortality rates [8,9]. Accurate estimates of adult tsetse mortality constitute an essential element of that understanding [10].

Laboratory animals and wild populations can differ greatly in their life expectancies [11]. To understand population dynamics in the wild, it is thus essential to obtain data from free-ranging field populations rather than laboratory animals [11]. Mark-recapture can provide good estimates of tsetse population parameters in closed situations, particularly where it is feasible to recapture the same flies many times during their lifetimes [12,13]. Mark-recapture studies are, however, logistically demanding, costly, and time consuming [10]. Moreover, in open populations subject to in- and out- migration, the results are often difficult to interpret, and researchers have accordingly developed alternate methods for estimating mortality [10,14].

As described more extensively elsewhere [15,16], ovarian dissection of tsetse can show the number of times a fly has ovulated. Since tsetse ovulate at approximately regular intervals, these data can be used to estimate the age distribution of female tsetse populations. It has been argued that it should then be possible, in principle, to determine how female mortality rates change with season, and over time, by analysing changes in age-distributions of female tsetse [17–20]. Current techniques for estimating female mortality from ovarian dissection data rely on three important assumptions [17]: first, that sampling probability is not dependent on the age of the fly; second, that mortality rates are independent of the age of the fly; third, and crucially, that the population under study has a stable age distribution.

A recent study shows, however, that these assumptions are often violated, leading to unrealistic mortality estimates. For example, standard techniques predict that mortality decreases with increasing temperature, which contradicts data from mark-recapture studies and is unlikely on biological grounds [10]. We therefore need new methods, for estimating female mortality from ovarian dissection data, which allow for unstable age distributions and for age-related changes in mortality and capture probability.

We develop dynamic models that simulate female tsetse populations, and the associated changes in their age distribution. At Rekomitjie, instability of the age distribution appears to result from large seasonal variation in temperature. Elsewhere, such instability could, however, result from other factors, such as seasonal changes in host density or rainfall. Our alternative approach to mortality estimation will be appropriate however the instabilities arise. Our models estimate or incorporate published estimates of temperature-dependent mortality in adults and pupae, temperature-dependent development rates in pupae, and density-dependent mortality in pupae [16,21,22].

## Methods

### Capture methods

The present paper is concerned with adult female *G*. *pallidipes* captured between 1 July 1991 and 30 June 1992 at Rekomitjie using stationary mechanical traps [23], baited with artificial host odour, consisting of acetone (dispensed at 500 mg/h), 1-octen-3-ol (0.4 mg/h), 4-methylphenol (0.8 mg/h) and 3-n-propylphenol (0.1 mg/h) [24]. The capture and processing methods have been described in detail elsewhere [5]. While fly populations and capture probabilities vary seasonally, trap catches were approximately the same at the start and end of the study period.

### Ovarian dissection

Female tsetse flies were subjected to ovarian dissection, and assigned to an ovarian category, depending on the relative sizes of the oocytes in the left and right ovaries [15,16]. For flies that have ovulated fewer than four times the ovarian category is equal to the number of times that the fly has ovulated. For flies that have ovulated more than three times, the ovarian category provides only the number of ovulations, or ovarian age, modulo four: that is, a fly in ovarian category 4 may have ovulated 4, 8, 12, 16 … etc. times, and analogous statements apply to flies in ovarian categories 5, 6 and 7. Flies too damaged to assign an ovarian category were excluded from the current analysis. During the study, 19,323 female *G*. *pallidipes* were dissected and assigned an ovarian category. The data were aggregated into monthly counts of flies in each category, as detailed in the Supporting Information.

### Temperature data

Daily maximum and minimum temperatures are routinely measured using mercury thermometers housed in a Stevenson screen at the station. We incorporated smoothed monthly mean temperatures (estimated as the monthly average of the maximum and minimum readings for each day of the month) in our model of female tsetse populations. Daily mean temperatures are given in the Supporting Information.

### Model

**Fig 1:**
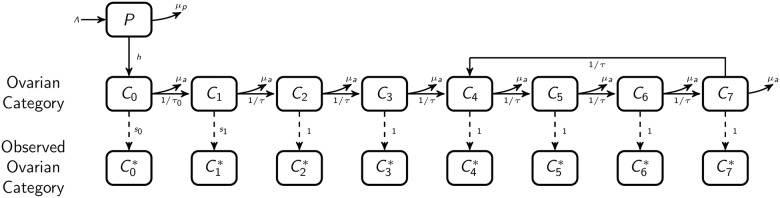
Model structure. Compartmental model for female tsetse populations. Pupae (*P*) are deposited at a rate *Λ*, proportional to the number of adult females in categories 1–7. Pupae die at a rate *μ*_*p*_, taken to be a function of pupal density and temperature. Pupae hatch at a rate *h*, becoming category 0 flies (*C*_0_). *C*_0_, *C*_1_, *C*_2_, and *C*_3_ correspond to flies that have ovulated 0,1, 2, and 3, times respectively, while *C*_4_ –*C*_7_ are aggregated categories of flies such that *C*_*x*_ for *x* = 4, 5, 6, or 7 corresponds to the total number of flies have ovulated *x+4n* times, where n is a whole number. Category 0 flies progress to category 1 at a rate of 1/6 per day. Category 1–7 (*C*_1_ –*C*_7_) flies progress to the next category at a rate of 1/9 per day, with category 7 flies progressing to category 4. All adult flies (*C*_0_ –*C*_7_) have a mortality *μ*_*a*_, a function of temperature. $C_{0}^{*}-C_{7}^{*}$ correspond to the number of flies available for sampling, i.e. susceptible to capture with traps; flies in category 0 and 1 flies are assumed to have lower probabilities of capture than older flies.

We develop a deterministic compartmental model of female tsetse populations based on an understanding of tsetse biology acquired from field and laboratory data. We model only the female population since this is the productive part of the population, for which we have age distribution data. Male and female *G*. *pallidipes* at Rekomitjie accrue mouthpart and mid-gut trypanosome infections at similar rates but, since females also live significantly longer than males, they are more likely to transmit trypanosomes (3). There are always sufficient *G*. *pallidipes* males at Rekomitjie such that about 98% of females are inseminated by the age of 8 days [16]. We do not, therefore, need to model the male population in order to model the dynamics of the female population. Our model assumes that all females are inseminated and ovulate at 6 days in age. The schematic of the model is shown in Fig 1 and parameters are described in Table 1. Tsetse could be in one of nine groups: the pupal stage and adult ovarian ages 0 to 7.

**Table 1:** Model parameters. Fitted and fixed model parameters.

Parameter Name	Parameter Description	Parameter Value
*S*_0_	Relative risk of capture for category 0 flies compared with category 2+ flies	fitted
*S*_1_	Relative risk of capture for category 1 flies compared with category 2+ flies	fitted
*α*	Parameterizes increase in immature mortality with temperature. See Eq 4. [per degree C]	fitted
*β*	Parameterizes increase in mature adult mortality with temperature. See Eq 3. [per degree C]	fitted
*μ*_*a*_	Per day mortality rate for adult female flies at temperatures less than 25°C [per day]	fitted
*d*	Parameterizes increase in pupal mortality with increasing pupal density. See Eq 1. [per pupa per day]	fitted
*a*	Parameterizes pupal hatching rate. See Eq 2.	5.5
*b*	Parameterizes pupal hatching rate. See Eq 2.	-0.25
*k*	Parameterizes pupal hatching rate. See Eq 2.	0.057
*μ*_*p*_	Minimum per day mortality rate for pupae [Instantaneous rate of deaths per day]	0.01
*τ*_0_	Time to first ovulation [days]	6
*τ*_1_	Time between successive ovulations [days]	9
*Λ*	Pupal deposition rate [pupae/day]	Proportional to number of adult females in categories 1–7

The assumed pattern of mortality among adult female tsetse is based on field experiments from Zimbabwe [17,18,21]. The experiments showed that adult female *G*. *m*. *morsitan*s suffered mortality in excess of 10% per day immediately after emergence: mortality then declined rapidly to about 3% by age 8 days, and then to about 1% per day at age 20 days. Thereafter, mortality increased steadily but slowly with age, such that the mortality was still less than 2.5% per day at age 100 days. Female adult mortality is thus clearly a function of age, but the major changes are restricted to the immature stages, while the fly’s thoracic musculature is still developing, and before it has ovulated for the first time. Notice that, since we are fitting our models only to ovarian age data from adult females, and we have no data on pupal numbers or losses, we cannot separate between deaths that occur in the pupal stage proper and those that occur among flies that have just emerged from the pupa and are not yet available to traps. Thus, while for convenience, we formally apply temperature and density-dependent mortality to pupae in the modelling, pupal mortality needs, strictly, to be interpreted as mortality in all stages prior to the mature adult stage. In subsequent text and tables, we refer to this mortality as immature mortality. We assumed, as a first approximation, that mortality was constant for all mature adult females. We also include, however, a model in which mature adult female mortality was allowed to vary with age in the Supporting Information.

Adults in a given age category are assumed to progress to the next age category at a constant rate, with flies of ovarian age 0 progressing to ovarian age 1 at a rate of (6 days)^-1^ and flies of all other ages progressing to the subsequent ovarian ages at a rate of (9 days)^-1^[6,7,16,25]. These rates are all temperature dependent but, at the field temperatures observed at Rekomitjie, the times between ovulations are unlikely to vary by more than 2 days from the assumed mean value and should not thus be a source of major error in the mortality estimates.

Pupae are deposited at a rate equal to the number of flies in ovarian categories 1 to 7 divided by the expected amount of time (9 days) which flies remain at these ovarian ages. Since only half of these pupae are female, this quantity is then divided by two to obtain the number of female pupae deposited. In other settings a pupal mortality of around 1% per day [26] has been assumed; in order to encompass this estimate in our models, we set the minimum pupal mortality rate below this at 0.001 per day. There is evidence for density-dependent mortality in pupae in the wild [26]: accordingly, we model a density-dependent pupal mortality rate proportional to the number of female pupae. The pupal mortality rate at temperatures under 25°C is given by:

$$\mu_{p,T\leq 25}=0.001+dP,$$ (1)

Where *d* parameterizes the density dependence and *P* is the pupal population in a given, but undefined, area.

Pupal duration is temperature-dependent in tsetse and female pupa emerge to become adults in age category zero. Laboratory studies show that pupal emergence rates and temperature are related by [27]:

h(T)=k/(1+exp(a+bT)) (2)

where *T* is the mean daily temperature and *k*, *a*, and *b* are constrained to be the values given in Table 1. We assume that Eq (2) also provides acceptable estimates of pupal duration for female *G*. *pallidipes* at Rekomitjie.

Mark-recapture studies suggest that mortality in adult female *G*. *pallidipes* increases exponentially with temperature (*T*) for *T* > 25°C [28]. Accordingly, we model adult fly mortality using:

$$\mu_{a}\left(T,T>25{}^{\circ}C\right)=\mu_{a,T\leq 25}\text{exp}\beta \left(T-25\right)$$ (3)

Where *μ*_*a*,*T*≤25_ is the mature adult mortality for temperatures at or below 25°C, and *β* parameterizes the increase at higher temperatures.

Pupal mortality is also thought to be temperature dependent and we also allow this quantity to increase exponentially with temperatures above 25°C:

$$\mu_{p}\left(T,T>25{}^{\circ}C\right)=\mu_{p,T\leq 25}\text{exp}\alpha \left(T-25\right),$$ (4)

Where *μ*_*p*,*T*≤25_ is the adult mortality rate for temperatures at or below 25°C given in Eq 1, and *α* parameterizes the increase in the mortality rate at higher temperatures.

Field evidence from Zimbabwe indicates the probability with which a female tsetse is captured in a trap increases with her age, with the greatest bias against flies in ovarian categories 0 and 1 [5,29]. We model this by allowing a relative risk of capture less than 1 for these young flies.

### Simulation and analysis

All simulation and analysis was performed using R 3.3.0. Differential equations specifying the three models, based on the compartmental model diagram in Fig 1, are given in the Supporting Information. Differential equations were solved numerically using the Livermore solver in the *deSolve* package. Since starting conditions were unknown, models were run for three years, which allows enough time for a stable pattern in population levels to be achieved. For the last year of the simulation, the average ovarian age distribution was calculated for each month of the study and compared with the observed ovarian age distribution for that month. For the main analyses, the log-likelihood for a given month was calculated assuming multinomial sampling of the available population. For the fits to both ovarian dissection and population data, the log-likelihood of the population data given the model was calculated using binomial sampling probabilities and added to the log-likelihood of the ovarian dissection data. To do this, catches per trap were scaled such that equal weight was given to both datasets, the true population was scaled such that it was large compared to the catch population (10,000 times on average), and the sampling probability was assigned to be the mean ratio of the scaled catch population to the true population. The overall log-likelihood was calculated as the sum of the log-likelihoods for each month. Since the population was roughly the same at the beginning and the end of the study period, a simulation was given a likelihood of zero if the simulation failed to produce a pupal population on July 1, 1991 within 3% of the pupal population on June 30, 1992.

Rates of pupal development, of transition between ovarian categories, and of minimum pupal mortality were fixed at the values suggested in the literature: all other parameters were determined using model fits. For each model, the maximum log-likelihood of the model was determined using the Nelder-Mead downhill-simplex algorithm, as implemented using the *optim* function in R. The optimization was performed to determine the optimum parameter set for that model, using a logit scale for some parameters (*S*_0_, *S*_1_, *μ*_*a*_) and a log scale for the remainder of the unknown parameters. The Hessian was calculated and then inverted to give the Fisher information matrix, which was used to obtain confidence limits for the variables. The confidence limits were then detransformed to obtain the confidence intervals for each parameter. Nested models that have an AIC at least 6 units larger than the best performing model were considered to confer a significantly worse fit than the best performing model (less than 0.05 times as probable as the best performing model) [30].

### Bias analysis

Standard techniques for estimating female tsetse mortality from ovarian dissection data are biased, especially during the hot-dry season (November to March) [10]. The bias associated with standard techniques varies with temperature variation: where, as in Zimbabwe, this variation is large, the bias will be most severe. Tsetse near the equator, where temperature variations are small, may have age distributions that are approximately stable—although it is possible other environmental factors may lead to nonstable age distributions in these settings [10,25]. To quantify this bias, using simulated data from our model together with our the maximum likelihood parameters, we estimated daily mortalities for the study period using standard techniques, as described elsewhere [15,17,26,29] and reviewed by Hargrove and Ackley [10].

Using maximum likelihood parameter estimates, we generated simulated female tsetse populations with varying magnitudes of temperature variation, but the same mean temperature. For a parameter *f*, varying between 0 and 1, we created counterfactual daily temperature *T*_*new*_(*f*) for each day based on the true temperature in Zimbabwe, *T*, as follows:

$$T_{new}\left(f\right)=f\left(T-\bar{T}\right)+\bar{T},$$ (5)

Where $\bar{T}$ is the mean annual temperature. We then determined the mean bias in mortality estimation using standard techniques as a function of *f*.

## Results

### Fitting to age distribution data

**Fig 2:**
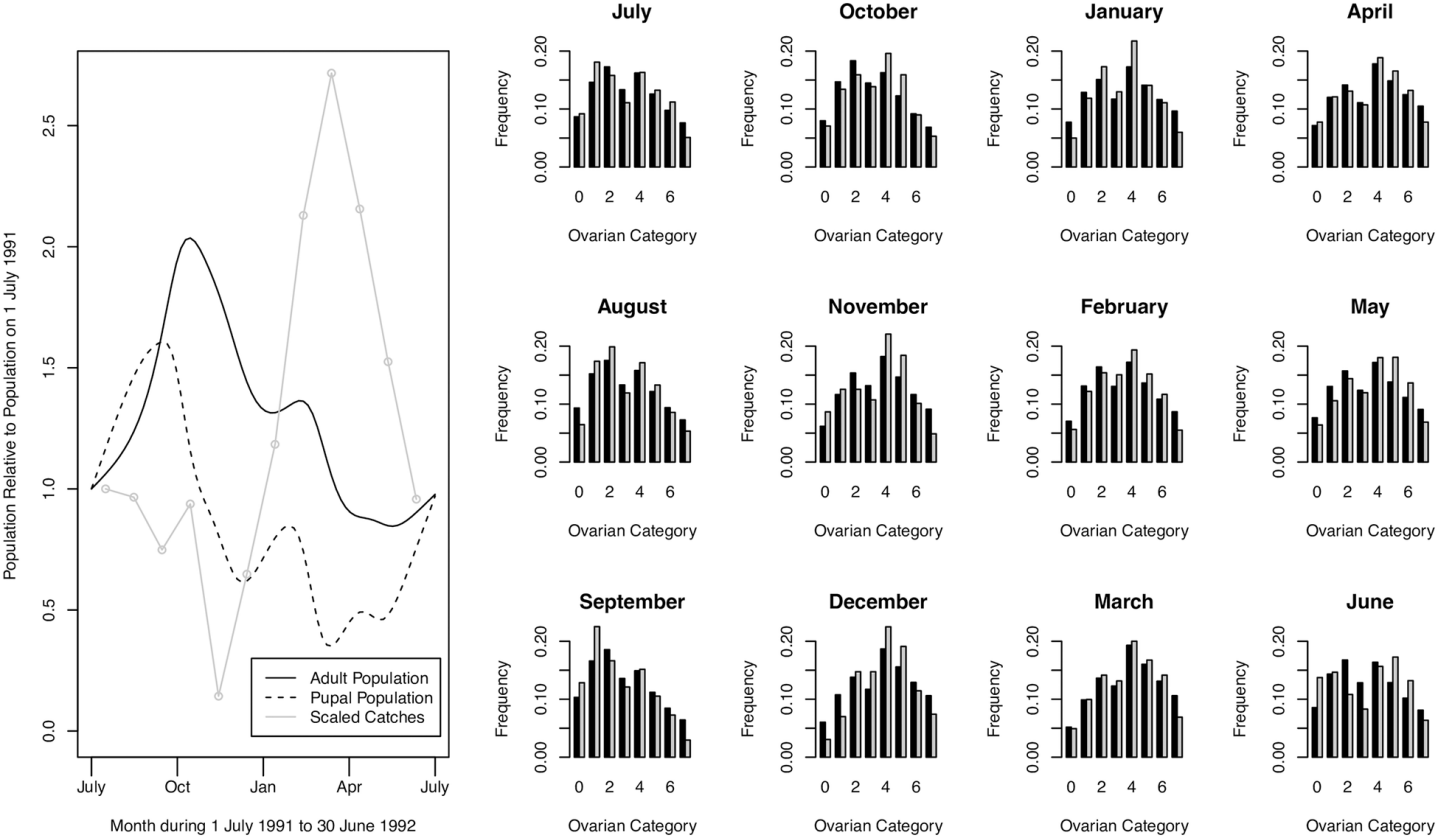
Monthly ovarian dissection data and maximum likelihood model fit for model 3. *Left*: Pupal and adult population over the course of a year for the maximum likelihood fit for model 3. Scaled trap catches are shown in grey. *Right*: Monthly ovarian dissection data and maximum likelihood model fit for model 3 shown in grey and black, respectively, for each month of the year 1 July 1991 to 30 June 1992. Fits for models 1 and 2 are given in the Supporting Information.

**Fig 3:**
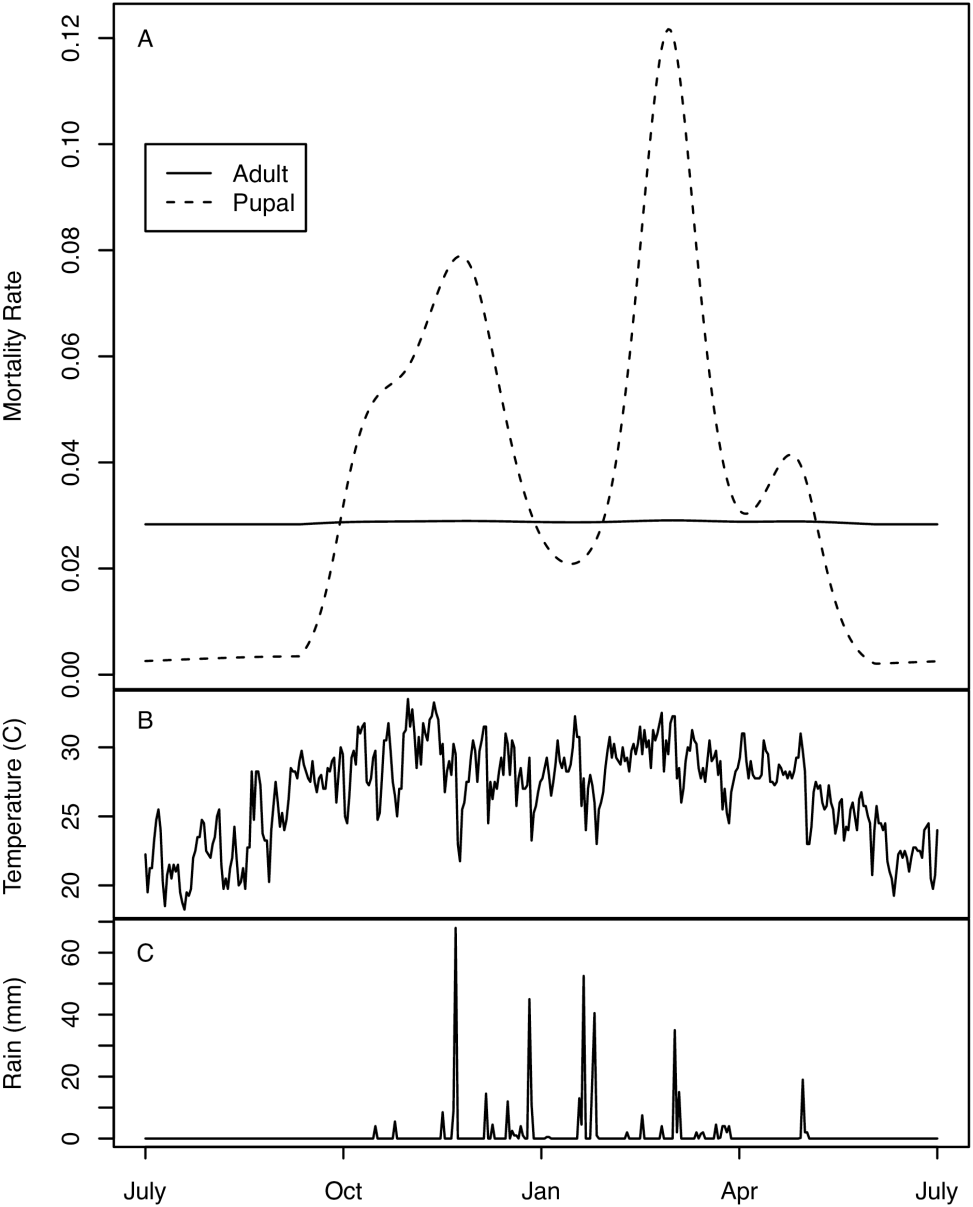
Estimated mortalities over the course of a year for model 3. Mortality rates among mature adults and immature stages over the course of the year estimated from the maximum likelihood fit for model 3 (A), mean daily temperatures (B), and daily rainfall (C). Mortality rates among immature stages vary much more with temperature, leading to peaks in mortality prior to, and following, the rainy season, with lower mortality among immature stages in the dry season between May and September.

**Fig 4:**
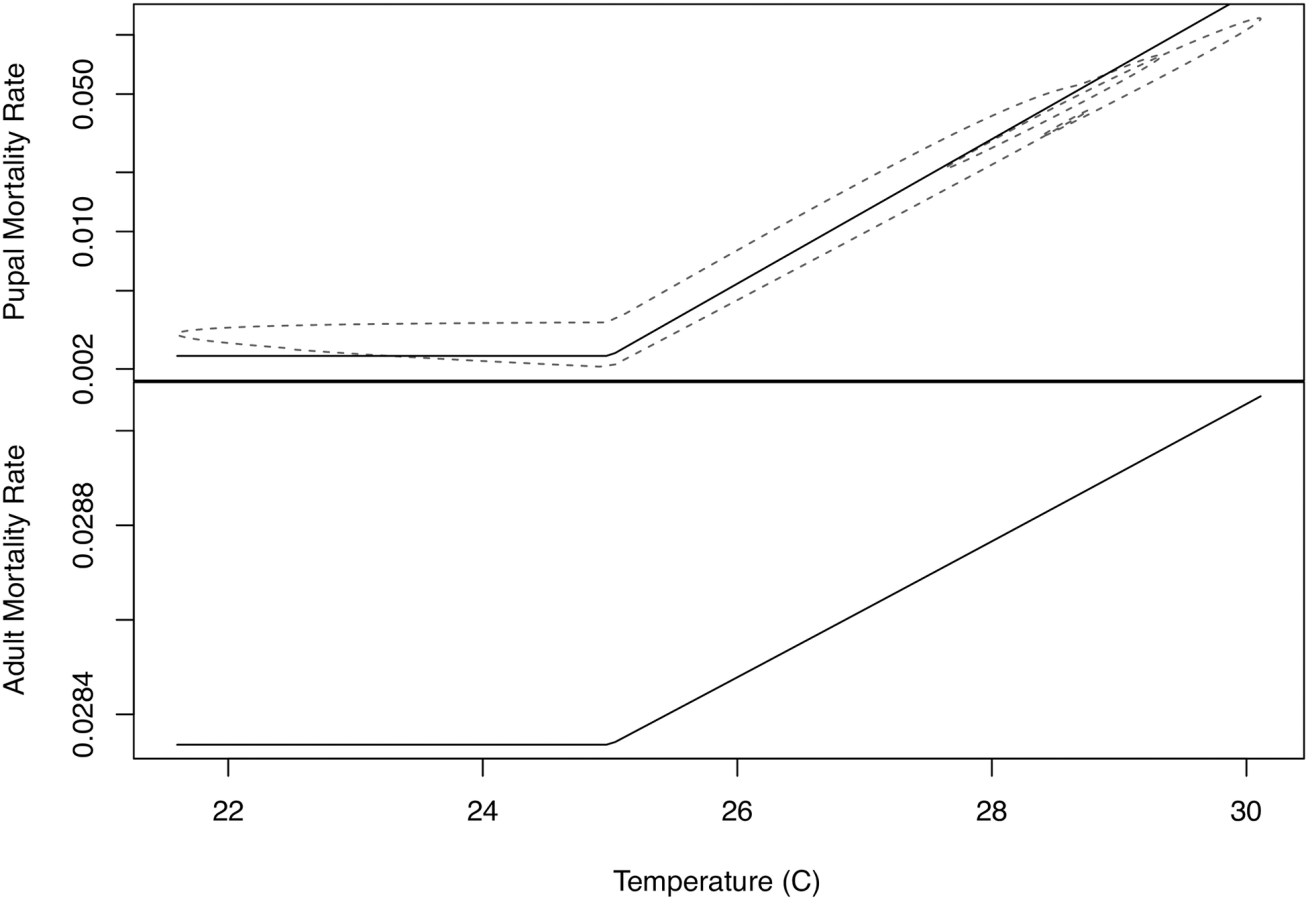
Estimated mortalities as a function of temperature for model 3. Estimated mortalities of immature stages (top) and mature adult females (bottom) as a function of temperature for the maximum likelihood fit for model 3. Mortality is plotted on a log-scale and note the differences in scale for the two graphs. Top: Mortalities are plotted for the estimated pupal population over the course of the year (dotted curve) and for the mean pupal population (solid line).

For the main analysis, we fit models 1–3 to the age distribution data, only constraining the population to be approximately the same at the beginning and end of the experimental period, but not taking any account of differences between predicted population levels and observed trap catches in the period between the endpoints. Maximum likelihood fits for model 3 provide a good fit to the age distribution data, but show large differences between predicted trends in simulated population and trap catches (Fig 2). Maximum likelihood fits are shown for models 1 and 2 in the Supporting Information. Table 2 summarizes the estimates of maximum likelihood parameters and their confidence intervals. Model 3, which allows for differential mortality between mature adults and immature tsetse, confers a significantly better fit than models 1 and 2. For all three models, the estimated mortality rate at temperatures less than 25°C is consistently 0.03 per day; 3% attrition per day is considered plausible for a non-decreasing tsetse population [12,31], and the maximum adult mortality before the population starts to decline is estimated to be approximately 4% per day [32]. For model 3, we estimate a mortality rate of 0.028 per day at temperatures less than 25°C, with mortality increasing for temperatures above 25°C. Figs 3 and 4 illustrate that mortality among mature adults hardly changes with time of year and temperature, whereas there is significantly more variation in the mortality of immature tsetse, which exhibit peaks in about November and March. Pupal mortality exceeds mature adult mortality except during the dry, generally cooler, months of May to September.

**Table 2:** Estimated parameters and confidence intervals from the dynamical model. Units in square brackets. Negative log-likelihoods (NLL) and Akaike Information Criterion (AIC) are given to one decimal place and parameter estimates are given to two significant figures. 95% confidence intervals are given in parentheses. Models. 3—Adult and pupal temperature dependent mortality: 2—Same temperature dependence for adults and pupae: 1—No temperature dependence.

Quantity	Description	Model 3	Model 2	Model 1
NLL/AIC	Negative log of the probability of the model given the data/Akaike Information Criterion	600.8/1213.7	835.3/1680.6	835.6/1679.1
*S*_0_	Relative risk of capture for category 0 flies compared with category 2+ flies	0.45 (0.42, 0.48)	0.43 (0.40, 0.46)	0.42 (0.39, 0.45)
*S*_1_	Relative risk of capture for category 1 flies compared with category 2+ flies	0.65 (0.62, 0.69)	0.63 (0.60, 0.66)	0.62 (0.59, 0.66)
*α*	Parameterizes increase in immature mortality with temperature. See Eq 4. [per degree C]	0.85 (0.75, 0.97)	0.0017 (0.00011, 0.025)	Constrained to be 0
*β*	Parameterizes increase in mature adult mortality with temperature. See Eq 3. [per degree C]	0.0050 (0.0012, 0.021)	Same as *α*	Constrained to be 0
*μ*_*a*_	Per day mortality rate for adult female flies at temperatures less than 25°C [per day]	0.028 (0.026, 0.031)	0.027 (0.026, 0.028)	0.027 (0.026,0.028)
*d*	Parameterizes increase in pupal mortality with increasing pupal density. See Eq 1. [per pupa per day]	1.4x10^-5^ (1.8x10^-8^, 1.0x10^-2^)	3.3x10^-5^ (7.0x10^-6^, 1.6x10^-4^)	3.5x10^-5^ (7.0x10^-6^, 1.7x10^-4^)

### Fitting age distribution data and trap catch data simultaneously

**Fig 5:**
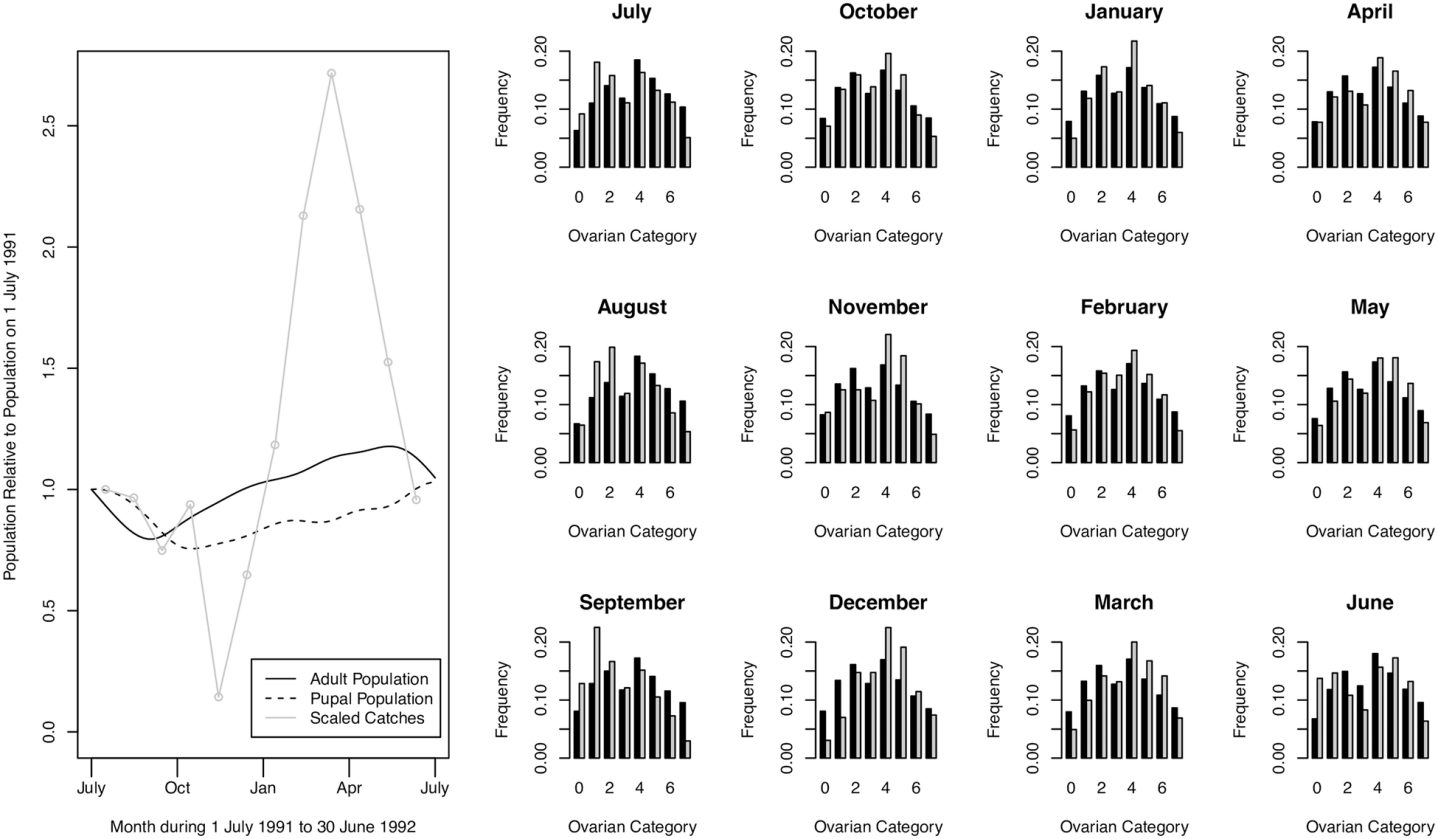
Monthly ovarian dissection data and maximum likelihood model fit for model 3 fit to both mean monthly trap catches and ovarian dissection data. *Left*: Pupal and adult population over the course of a year for the maximum likelihood fit for model 3. Scaled trap catches are shown in grey. *Right*: Monthly ovarian dissection data and maximum likelihood model fit for model 3 shown in grey and black, respectively, for 1 July 1991 to 30 June 1992.

When model 3 was fit to both the ovarian dissection data and the catch data simultaneously there was a poorer fit to the ovarian dissection data, as apparent in the considerably lower likelihood and from examination of the fits (Table 3, Fig 5). Using this model, it is not possible to simultaneously obtain good fits to both the age distribution and the trap catch data. Nonetheless, fitting to both data sources produces a mortality rate estimate for mature adults of 0.027 per day at temperatures under 25°C, comparable to estimates from the models fit only to the ovarian dissection data (Table 2). Given the challenges in fitting both data sources and the uncertainty of the correspondence between trap catches and population levels, we used model fits to only the age distribution data for further investigations.

**Table 3:** Estimated parameters and confidence intervals from dynamical model 3 fit to catch data as well as ovarian dissection data. Units in square brackets. Negative log-likelihoods (NLL) and Akaike Information Criterion (AIC) are given to one decimal place and parameter estimates are given to two significant figures. 95% confidence intervals are given in parentheses.

Quantity	Description	Value
NLL*	Negative log of the probability of the model given the ovarian data only.	837.9
NLL/AIC	Negative log of the probability of the model given all fitted data/Akaike Information Criterion.	3294.3/6600.5
*S*_0_	Relative risk of capture for category 0 flies compared with category 2+ flies	0.48 (0.45, 0.51)
*S*_1_	Relative risk of capture for category 1 flies compared with category 2+ flies	0.66 (0.63,0.70)
*α*	Parameterizes increase in immature mortality with temperature. See Eq 4. [per degree C]	0.013 (0.013, 0.013)
*β*	Parameterizes increase in mature adult mortality with temperature. See Eq 3 [per degree C]	0.0013 (0.0012, 0.0013)
*μ*_*a*_	Per day mortality rate for adult female flies at temperatures less than 25°C [per day]	0.027 (0.026, 0.027)
*d*	Parameterizes increase in pupal mortality with increasing pupal density. See Eq 1. [per pupa per day]	3.2x10^-5^ (2.9x10^-5^, 3.6x10^-5^)

### Estimation of bias in standard mortality estimation technique

**Fig 6:**
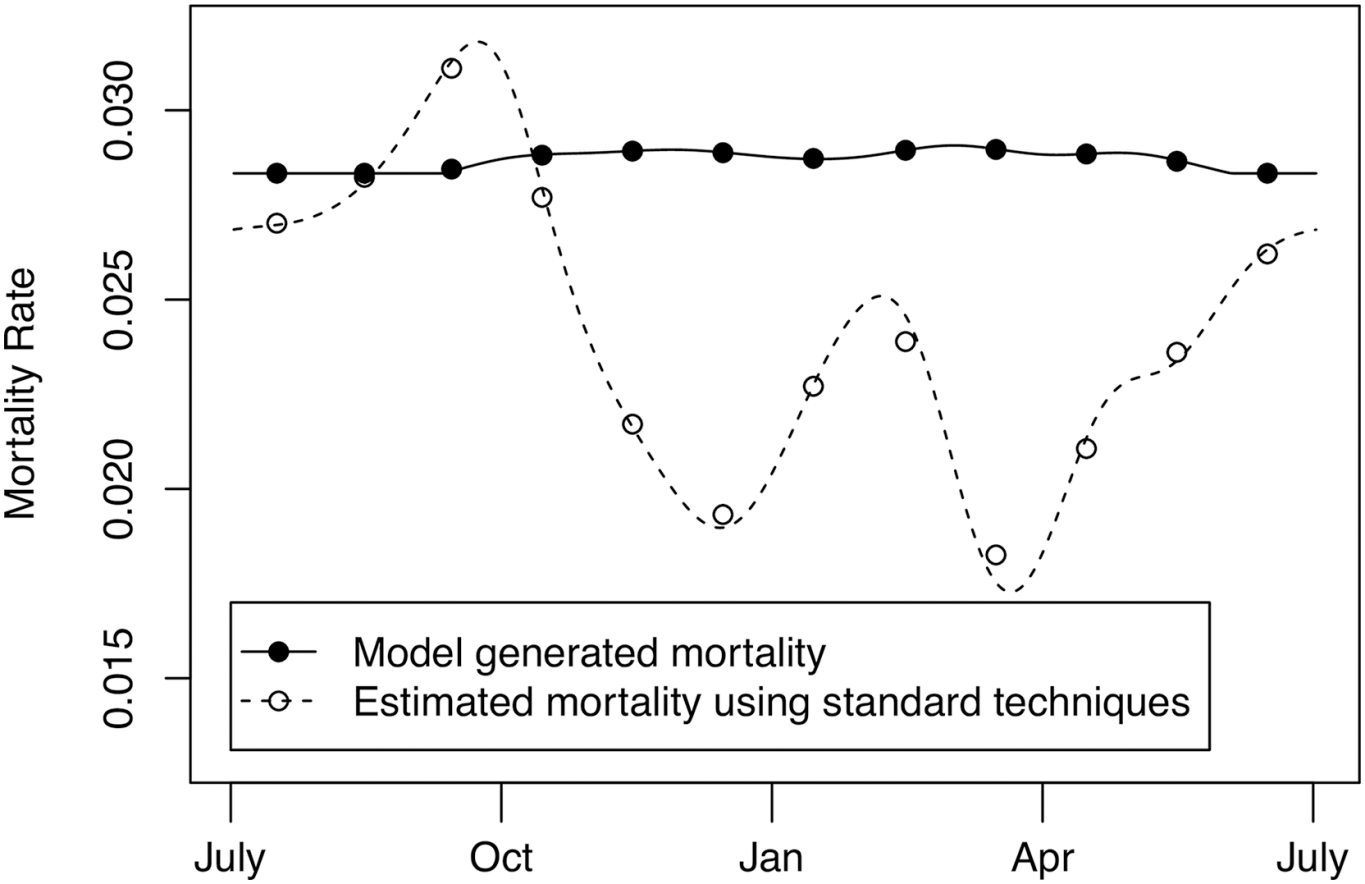
Estimated bias of standard mortality estimation. Mortality estimates using standard techniques, or simulated using maximum likelihood parameter estimates from model 3. We assume that mortality estimation was performed with daily ovarian dissection data (lines) and monthly ovarian dissection data (dots). Estimates are most biased during the hot-dry seasons, when standard techniques give the lowest mortality estimates while the true mortalities are at their highest.

We used model 3, the minimum AIC model in our main analysis, and corresponding parameter estimates (Table 2) to quantify the possible biases inherent in standard mortality estimation techniques. We generated daily ovarian category distributions using observed smoothed monthly mean temperatures at Rekomitjie. With these distributions, we estimate the mortality among mature adult tsetse for that day. Fig 6 shows the model-generated mortality (solid line) and the mortality estimated using standard techniques (dashed line) as a function of time of year at Rekomitjie, assuming the maximum likelihood parameter estimates for model 3 (Table 2). Standard techniques are biased at all times of year, but the bias is largest during the hot-dry season. In this season, standard techniques give the lowest mortality estimates when mortality is in fact highest. Our modelling suggests, however, that this increased mortality occurs almost exclusively among recently emerged, immature, adults. Since data are typically aggregated by month to estimate a monthly mortality, Fig 6 also shows the monthly mean of mortalities generated using our model (closed dot) and estimated using standard techniques (open dot). Aggregating the data by month does not significantly alter the magnitude of the bias.

**Fig 7:**
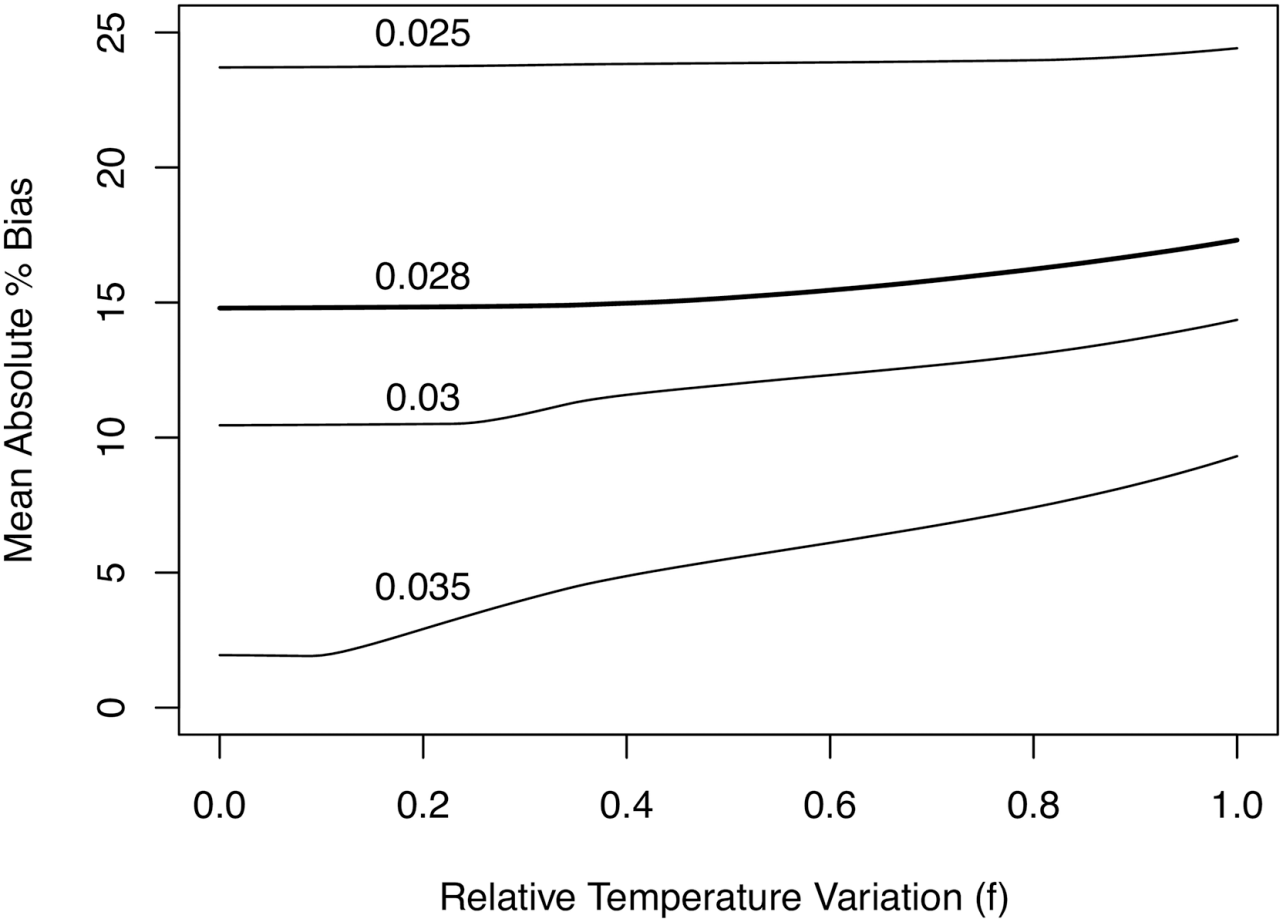
Mean bias from standard mortality estimation techniques. Mean bias from standard mortality estimation techniques using data simulated from various adult fly mortalities (text annotations) and maximum likelihood parameter estimates for the remainder of the parameter from model 3 as a function of temperature variation. *f = 0* corresponds to no temperature variation, and *f = 1* corresponds to the temperature variation at Rekomitjie.

Fig 7 shows the mean bias of standard mortality estimation techniques as a function of temperature variation. This bias is plotted for various mortalities (text annotations), using model 3 and the maximum likelihood parameter estimates for the remaining parameters. As shown in Eq 5, *f = 0* corresponds to no temperature variation, and *f = 1* corresponds to the temperature variation at Rekomitjie. The general trend is that bias in standard techniques is greater when there is greater temperature variation. However, the magnitude of the bias and the extent to which it depends on temperature variation depends on the mortality rate.

## Discussion

A major finding of this study is that mortality in immature tsetse is higher, and increases much more rapidly with increasing temperature, than in mature adults. These results are consistent with results from a mark-recapture study showing that mortality among recently emerged female tsetse is markedly higher than for all older flies [16,21]. It may be objected, however, that the high mortality estimated in the mark recapture experiment, in newly emerged—and newly marked and released—flies might merely reflect stress due to their handling and marking. This possibility was acknowledged in the original analysis [33], but it was argued that the continual decrease in the loss rate over the first 18 days of life was consistent with high (natural) losses in young flies. This conclusion is also consistent with published evidence that a large percentage of newly emerged tsetse can die before they become available for capture in the field and that this percentage can increase dramatically at high temperatures [31,33–35].

The results are, admittedly, at variance with published data on other insects where there have been no reports of mortality being higher in recently emerging insects than in older adults. In part, however, this may reflect the difficulties attendant on estimating insect mortality in the field. The case of age-dependent mortality in mosquitoes provides a good example of the problems involved. The following analysis of published methods for estimating age-specific mortality in mosquitoes suggests that they would not allow the detection of increased mortality in newly emerged field mosquitoes even if it existed.

To our knowledge, nobody has yet carried out on mosquitoes, or indeed on any other insect, the equivalent of the experiment on tsetse where insects were marked uniquely, released in field at birth, and where their recapture history was recorded for the rest of their lives [16,21]. Early analysis of the age structure of mosquitoes captured in the field showed that mortality rates increased with age [36]. There was no evidence for increased mortality in the youngest mosquitoes, but this method obviously cannot estimate the number of mosquitoes that die before they become available for sampling and thus cannot provide any estimate of mortality among the youngest mosquitoes.

This objection does not apply to a study where the survival of large samples (>10,000) of mosquitoes was followed from birth [37]. For both sexes, mortality was low at young ages (< 10 days old), steadily increased among middle-aged mosquitoes, and decelerated at older ages. Again, therefore, this experiment produced no evidence for increased mortality among young mosquitoes. However, this was a study of laboratory-bred and raised mosquitoes and, again, says nothing about the rate at which newly emerged mosquitoes might die in the field. As observed above and elsewhere, mortality among young tsetse in the laboratory is much lower than estimated for field flies [21]: the same may well be true for mosquitoes.

The “captive cohort method” for estimating population age structure in the wild can be used to estimate age-specific mortality rates. Since, however, the method involves following the survival of samples captured in the field [38–40], the problem referred to above arises: the method cannot provide mortality estimates for insects that die before they can be sampled. Moreover, while the method is valid for stationary populations (stable age distribution and zero growth rate), violations of this assumption require more complex modelling approaches, with quantification of population birth rates or immigration/emigration rates [39].

Thus, while the studies reviewed here produced no evidence for increased mortality in very young mosquitoes, the methods used would not anyway be able to detect increased mortality among newly emerged mosquitoes in the field. Nonetheless, it is not unreasonable to expect that the risks faced by young tsetse, relative to mature adults, are much greater than those faced by their mosquito counterparts. Teneral tsetse have low levels of fat, poorly developed flight musculature, relatively weak flight capability and, being obligate blood feeders, need to locate a vertebrate host and feed off it safely before they starve [41–43]. The problems for newly emerged mosquitoes are less severe: the flight performance of *Aedes aegypti*, for example, is highest during the first 14 days of life [44]: since, also, mosquitoes can feed off nectar and plant juices, their feeding risks should be much lower than for tsetse.

Since we were only fitting our models to age distributions of adult females, we had no way of separating death rates in immature tsetse between deaths that occurred in the pupal stage and those occurring in very young adults, before they became available for trap capture. Regardless of how these deaths among immature classes are counted, however, if they occur in large numbers they will contribute to destabilisation of the population age structure. This effect has bedevilled past efforts to estimate female tsetse mortality from ovarian dissection data, which assumed that the population age distribution was stable [10]. We developed dynamic models that allow for age distributions that are unstable, in our case due to fluctuations in temperature, and also allow for age-related changes in mortality and capture probability.

For each of the three models, mortality estimates are around 0.03 per day for temperatures under 25°C, consistent with estimates from other areas. Mark-recapture studies at the nearby Antelope Island gave an adult female mortality rate of 0.023 at lower temperatures, but with a larger increase in mortality with increasing temperature than predicted by model 3 [33]. Previous mark-recapture work has shown that the increase in mortality for adult *G*. *pallidipes* females at higher temperatures is exponential and characterized by a coefficient of 0.106 [33], which is much larger than the value of the analogous parameter (*β*) estimated for model 3. The disagreement could be due to the incorporation in the mark-recapture estimates of some young flies that still have higher natural mortality rates than fully mature flies. Mortality is highest among flies that have just emerged, but only declines to mature levels over the first 10 days of adult life [33].

Our modelling produces mortality estimates that appear more reliable than those from classical analyses in that mortality is predicted to increase with temperature, as expected. Our technique also offers insight into the factors driving the dramatic changes in age distribution observed during the hot-dry season. Model 2, where mortality among mature adults and all immature tsetse have the same dependence on temperature, does not perform significantly better than model 1, where mortality is independent of temperature. Temperature-dependent mortality for immature stages and mature adults cannot thus explain the observed changes in age distribution if it is applied evenly across all stages. Model 3, which allows for differential dependence of mortality on temperature for mature adults and immature stages, performs significantly better than models 1 and 2. This differential dependence of mortality on temperature causes the observed ratio of young flies to older flies to decrease at high temperatures, which would explain why a greater fraction of flies sampled during the hot-dry season are older than at other times of year.

Our results suggest that increases in temperature affect the mortality of recently emerged adult *G*. *pallidipes* more than all older flies. Age-dependent mortality has been documented in the field for *G*. *m*. *morsitans* [21] and in the laboratory for various tsetse species [22,45,46], and we suggest that these differences may not remain constant with changes in temperature. Using standard mortality estimation techniques to estimate fly mortality can demonstrate the bias inherent in these techniques and show how this bias varies seasonally for locations near and far from the equator. We find that during the hot-dry season, the mortality estimates using standard techniques are most biased, whereas during the rest of the year, the bias is smaller, though still significant (Fig 5). We also find that the bias in standard techniques will tend to decrease with decreasing annual variation in temperature. Standard techniques, which assume a population that is declining or growing exponentially [17,29], may nonetheless give biased mortality estimates, and the magnitude of this bias is not easily predicable since it may depend on parameters such as the mature adult mortality. An unpredictable bias can make it impossible to compare mortalities at different times or in different regions.

Elsewhere in Africa, particularly at sites close to the Equator, it may be true that temperature variations are not sufficient to produce age structure instability. However, the possibility cannot be excluded that age structure instability could result elsewhere from other climatological effects: any such effects will cause errors in mortality estimates where these are derived using the classical approach. It is thus incumbent on investigators to convince themselves whether or not their study population does indeed exhibit a stable age structure.

Our work has several strengths: (i) Extension of traditional mortality estimation techniques from ovarian dissection data. Our model is an extension of traditional mortality estimation techniques from ovarian dissection data that allows for non-stable age distributions and explicitly constrains population growth. This allows us to make mortality estimates that are directly comparable to those from traditional techniques, while avoiding incorrect assumptions. (ii) Dynamical modelling. Dynamical modelling techniques, such as the compartmental modelling we employ, have been used to estimate key demographic and disease transmission parameters in many settings, including for vector populations [47] and tsetse populations [12]. (iii) Model based on an understanding of tsetse biology acquired from field and laboratory data. Our models include temperature-dependent pupal emergence, pupal (and immature) mortality, and mature adult mortality, as well as density-dependent pupal mortality.

Our work also has several limitations. (i) Unknown populations of adults and pupae over the course of the year. The most serious challenge we have in our modelling arises from our ignorance regarding the way in which the true population numbers of adult and puparial tsetse vary with time and season. Differences in tsetse trap catches from month to month are undoubtedly related to population changes; however, they also plausibly reflect changes in fly behaviour, and thus capture probability, with changing temperature, and potentially reflect changes in age structure and seasonal in- and out-migration [48]. For our main analysis, we did not constrain our models to fit population numbers, whether for adult or immature stages. The different models presented in Tables 2 and 3 give very different estimates for how the pupal and adult fly populations change over the course of the year. Nonetheless, the mortality estimates for temperatures under 25°C did not vary significantly between the three models, and, for model 3, with or without fitting to trap data. The mortality estimates for temperatures less than 25°C do not, therefore, appear to be particularly sensitive to the way in which the total population is predicted to change. However, more accurate estimates of population changes over the course of the year would be required to determine how high temperatures affect mortality.

(ii) Classification of teneral deaths. As detailed in the Methods section, we could not separate pupal deaths from those occurring in newly emerged adults before they could be trapped. Indeed, it is not always clear how to categorise deaths among immature classes. Pupae that are predated or parasitized are clearly true pupal deaths. A major loss of immature flies is, however, due to the excessive use of fat at extreme temperatures during the pupal phase [34]. If so much fat is used during the pupal phase that the emerging fly has insufficient energy to find its first meal—or even fails to emerge—it is unclear whether this should be characterised as a pupal or a teneral adult death. For convenience, we have modelled temperature-related deaths in immature stages as occurring with equal probability across the pupal stage but, in reality, most of these deaths will actually only occur at, or very shortly after, emergence. In addition, at high temperatures, the abortion rate increases to about 2% and the high pupal mortalities produced by our model fit may be accounting for this as well [49].

(iii) Assumptions about the pupal mortality rates. We assumed a density dependence of a specific form with a minimum pupal mortality of 0.001. Since the maximum likelihood estimate of the pupal population was always large enough such that the pupal mortality was much greater than this minimum, our model was not sensitive to this assumption. Previous research has indicated that pupal mortality increases sharply for temperatures < 18°C [50]. During the period of our study, however, the 20-day running mean temperature at Rekomitjie never dropped below 20°C. Accordingly, for simplicity, we only model increases in pupal mortality with increasing temperature (see the Supporting Information).

(iv) Age independent mortality for mature adult females. We do not include age-dependent mortality for adults in models 1, 2, or 3. While mortality does increase with age among mature adult female tsetse, our dynamic models capture the spirit of the field-estimated changes in mortality with age among female tsetse in having: (i) very high mortality in immature adults (inasmuch as a high pupal mortality approximates this); (ii) low mortality in mature adults; (iii) a rate of increase in mortality with age that is very small—and in fact taken as zero in our model. As an additional sensitivity analysis, we have included in the Supporting Information a model where mortality among mature adults is allowed to vary with age. This model produces similar mortality estimates to those produced by model 3, and also predicts a very low rate of increase in mortality with the age of mature adult females.

(v) Other factors omitted from the model. Tsetse biology is complicated and our models did not include every possible factor that could affect mortality estimation. For example, we assumed that the time taken for a female to produce her first larva, and the subsequent interlarval periods, were independent of temperature. This is known not be the case at Rekomitjie, but the variation amounts only to a few days over the whole temperature range and errors arising from this approximation may be expected to be small relative to other errors. In addition, meteorological factors other than temperature may affect tsetse mortality and these factors were not included in the model. The inclusion of additional factors could help to address the difficulty in obtaining good fits to catch and ovarian data simultaneously.

In conclusion, we developed a dynamical model that produces adult tsetse mortality estimates and temperature-dependent increases in mortality consistent with mark-recapture studies. Mortality estimates at temperatures under 25°C are consistent across a number of sensitivity analyses. This model provides important insights into the changing ovarian age-distribution over the course of the year: differential increases in mortality for pupae and young adults versus older adults lead to higher mean ovarian ages at the hottest times of the year. In addition, standard techniques for mortality estimation may be highly problematic in areas with large variation in temperature, such as Rekomitjie. In areas near the equator with less marked variations in temperature, standard techniques may still be significantly biased. Lastly, with longitudinal ovarian dissection data, or indeed any age distribution data, the dynamic modelling approach we employ could be used to estimate mortalities in other settings, and could be modified to answer other research questions, such as the effects seasonal changes in host density.

## Supporting information

S1 Fig Monthly ovarian dissection data and maximum likelihood model fit for model 1. *Left*: Pupal and adult population over the course of a year for the maximum likelihood fit for model 1. *Right*: Monthly ovarian dissection data and maximum likelihood model fit for model 1 shown in grey and black, respectively, for July 1991 to June 1992.
(TIF)

S2 Fig Monthly ovarian dissection data and maximum likelihood model fit for model 2. *Left*: Pupal and adult population over the course of a year for the maximum likelihood fit for model 2. *Right*: Monthly ovarian dissection data and maximum likelihood model fit for model 2 shown in grey and black, respectively, for July 1991 to June 1992.
(TIF)

S3 Fig Mean catches per trap. Mean catches per trap for 1 July, 1991 to 30 June 1992.
(TIF)

S1 Data Ovarian dissection and temperature data used in this study. (DOCX)

S1 Additional Models Additional models included as sensitivity analyses. (DOCX)

S1 Equations Equations specifying the dynamical model. (DOCX)
