# Supplementary figures and images for: A dynamic model for estimating adult female mortality from ovarian dissection data for the tsetse fly Glossina pallidipes Austen sampled in Zimbabwe
Source: PLoS Negl Trop Dis. 2017 Aug 30;11(8):e0005813. doi: 10.1371/journal.pntd.0005813 (PMC5576662; doi:10.1371/journal.pntd.0005813)

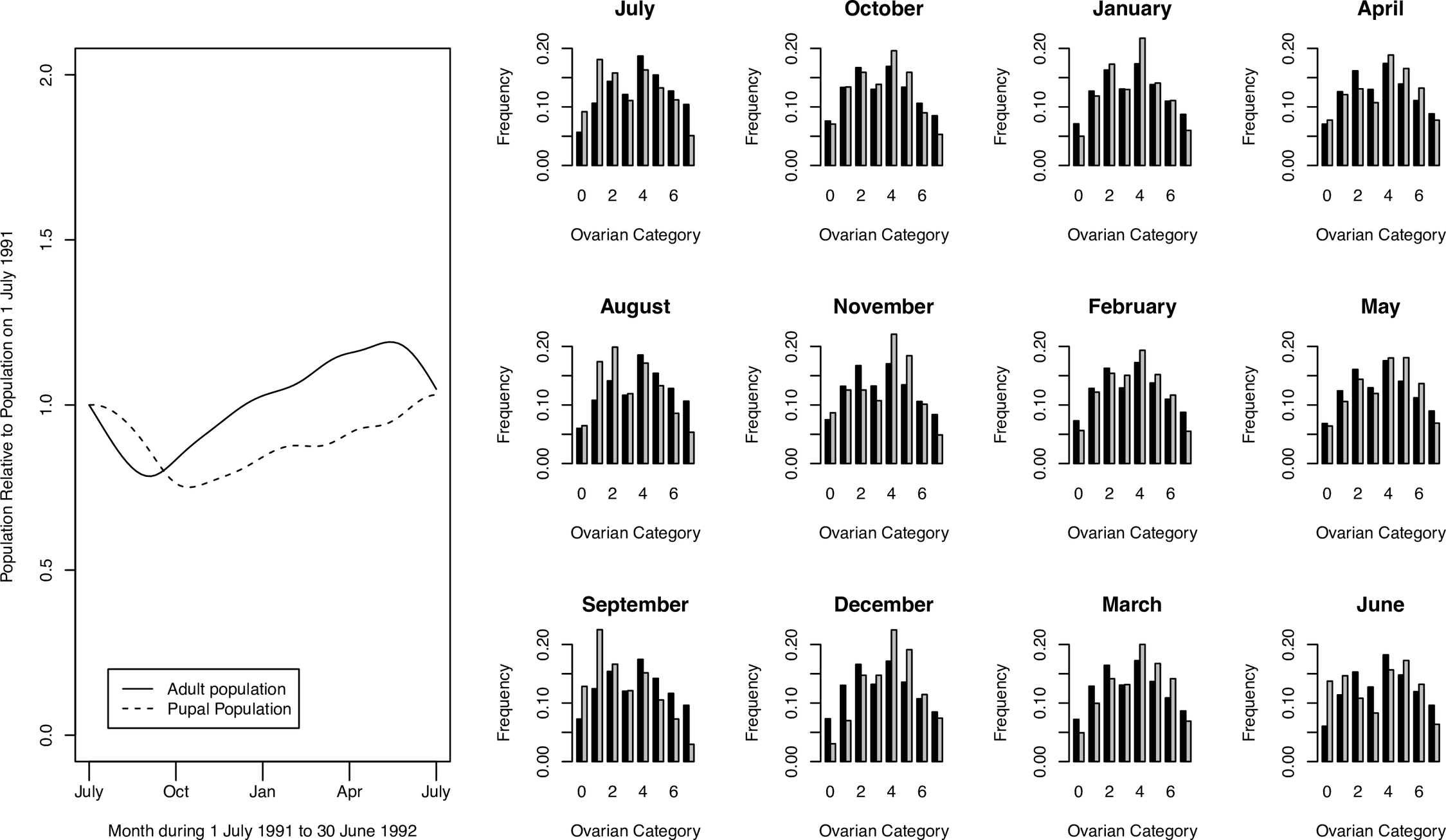

Supplement: S1 Fig — Left: Pupal and adult population over the course of a year for the maximum likelihood fit for model 1. Right: Monthly ovarian dissection data and maximum likelihood model fit for model 1 shown in grey and black, respectively, for July 1991 to June 1992. (TIF) [file pntd.0005813.s001.tif]

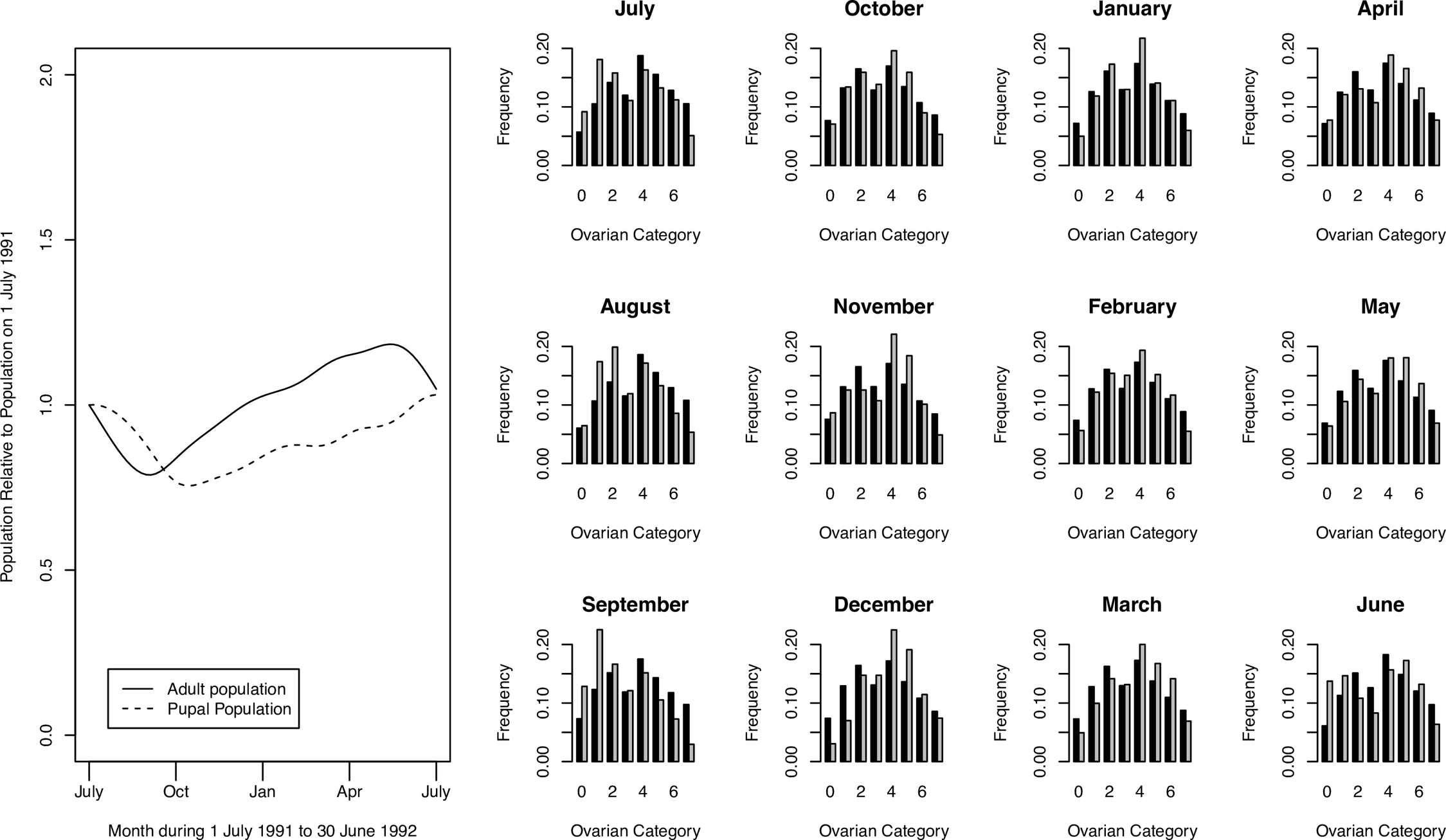

Supplement: S2 Fig — Left: Pupal and adult population over the course of a year for the maximum likelihood fit for model 2. Right: Monthly ovarian dissection data and maximum likelihood model fit for model 2 shown in grey and black, respectively, for July 1991 to June 1992. (TIF) [file pntd.0005813.s002.tif]

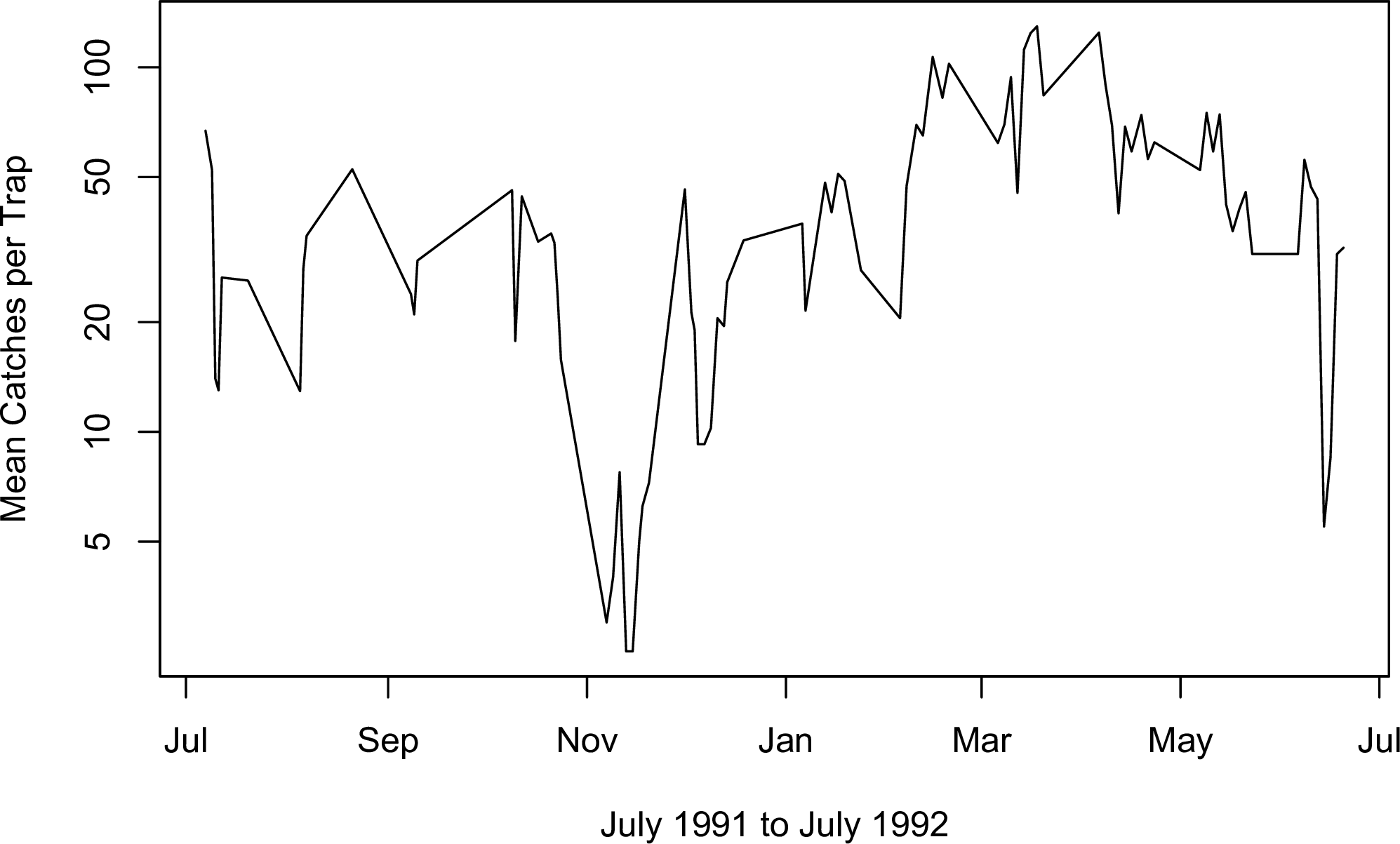

Supplement: S3 Fig — Mean catches per trap for 1 July, 1991 to 30 June 1992. (TIF) [file pntd.0005813.s003.tif]
