# Supplementary material for: A dynamic model for estimating adult female mortality from ovarian dissection data for the tsetse fly Glossina pallidipes Austen sampled in Zimbabwe
Source: PLoS Negl Trop Dis. 2017 Aug 30;11(8):e0005813. doi: 10.1371/journal.pntd.0005813 (PMC5576662; doi:10.1371/journal.pntd.0005813)
Supplement: S1 Data — (DOCX) [file pntd.0005813.s004.docx]

**S1 Data**

**Table A: Ovarian Dissection Data for July 1991 to June 1992**

|  | Ovarian Category | | | | | | | |
| --- | --- | --- | --- | --- | --- | --- | --- | --- |
|  | **0** | **1** | **2** | **3** | **4** | **5** | **6** | **7** |
| **July** | 72 | 142 | 124 | 87 | 128 | 104 | 88 | 40 |
| **August** | 52 | 140 | 160 | 96 | 138 | 107 | 69 | 43 |
| **September** | 122 | 214 | 158 | 115 | 144 | 100 | 69 | 28 |
| **October** | 48 | 91 | 108 | 94 | 133 | 108 | 61 | 36 |
| **November** | 71 | 103 | 103 | 88 | 181 | 151 | 83 | 40 |
| **December** | 37 | 85 | 179 | 179 | 273 | 232 | 139 | 90 |
| **January** | 45 | 107 | 156 | 117 | 196 | 127 | 100 | 54 |
| **February** | 133 | 288 | 364 | 356 | 457 | 359 | 276 | 130 |
| **March** | 186 | 376 | 535 | 497 | 756 | 633 | 535 | 261 |
| **April** | 252 | 394 | 426 | 349 | 614 | 539 | 430 | 252 |
| **May** | 158 | 261 | 355 | 295 | 445 | 446 | 337 | 170 |
| **June** | 179 | 191 | 141 | 108 | 204 | 225 | 172 | 83 |

**Table B: Daily Mean Temperature for 1 July 1991 to 30 June 1992**

|  | Jul-91 | Aug-91 | Sep-91 | Oct-91 | Nov-91 | Dec-91 | Jan-92 | Feb-92 | Mar-92 | Apr-92 | May-92 | Jun-92 |
| --- | --- | --- | --- | --- | --- | --- | --- | --- | --- | --- | --- | --- |
| 1 | 22.25 | 23.00 | 24.25 | 25.00 | 32.75 | 27.50 | 28.50 | 29.25 | 32.25 | 31.00 | 23.00 | 24.00 |
| 2 | 19.50 | 23.50 | 25.25 | 24.50 | 31.00 | 29.75 | 29.25 | 30.25 | 27.75 | 31.00 | 23.00 | 25.75 |
| 3 | 21.25 | 25.00 | 24.00 | 26.50 | 28.50 | 30.50 | 28.00 | 29.25 | 28.50 | 29.00 | 24.25 | 24.50 |
| 4 | 21.25 | 25.50 | 24.75 | 29.25 | 30.75 | 31.50 | 26.50 | 29.00 | 26.00 | 27.75 | 26.75 | 24.50 |
| 5 | 23.25 | 21.50 | 26.25 | 29.75 | 28.75 | 31.50 | 27.50 | 28.75 | 27.00 | 29.00 | 27.50 | 24.00 |
| 6 | 24.75 | 19.75 | 28.50 | 28.75 | 31.75 | 24.50 | 28.75 | 30.00 | 29.00 | 28.00 | 27.00 | 24.50 |
| 7 | 25.50 | 20.50 | 28.25 | 31.50 | 31.00 | 27.50 | 30.50 | 29.00 | 30.00 | 27.75 | 27.25 | 21.75 |
| 8 | 24.00 | 19.75 | 28.25 | 31.00 | 30.50 | 26.25 | 29.00 | 29.25 | 29.75 | 27.75 | 26.00 | 21.00 |
| 9 | 20.25 | 21.25 | 27.75 | 31.50 | 32.00 | 27.50 | 28.50 | 28.25 | 31.25 | 27.75 | 25.50 | 20.50 |
| 10 | 18.50 | 22.00 | 29.00 | 31.75 | 32.25 | 27.00 | 29.25 | 29.75 | 30.50 | 28.00 | 25.75 | 19.25 |
| 11 | 20.75 | 24.25 | 29.75 | 27.50 | 33.25 | 28.25 | 28.25 | 30.25 | 30.25 | 30.50 | 27.25 | 20.75 |
| 12 | 21.50 | 22.00 | 28.75 | 27.25 | 32.50 | 29.25 | 28.25 | 29.50 | 28.50 | 29.75 | 26.00 | 22.25 |
| 13 | 20.50 | 20.00 | 28.25 | 27.75 | 32.00 | 28.00 | 28.75 | 30.25 | 27.75 | 29.50 | 25.50 | 22.50 |
| 14 | 21.50 | 20.25 | 27.75 | 29.25 | 29.50 | 31.00 | 30.00 | 31.50 | 28.50 | 27.50 | 23.75 | 22.00 |
| 15 | 21.00 | 21.25 | 27.50 | 29.75 | 30.25 | 30.25 | 32.25 | 29.50 | 27.50 | 27.50 | 24.50 | 22.50 |
| 16 | 21.50 | 19.75 | 29.00 | 24.75 | 26.75 | 28.00 | 30.75 | 31.25 | 29.00 | 27.25 | 26.00 | 22.00 |
| 17 | 19.50 | 22.75 | 27.50 | 25.25 | 28.25 | 30.50 | 30.75 | 30.00 | 30.50 | 27.50 | 26.25 | 21.00 |
| 18 | 18.75 | 22.75 | 26.75 | 27.75 | 29.00 | 30.00 | 25.75 | 30.50 | 29.00 | 28.75 | 23.25 | 22.00 |
| 19 | 18.25 | 28.25 | 27.75 | 30.50 | 28.00 | 25.75 | 27.75 | 28.50 | 29.25 | 28.50 | 24.25 | 22.75 |
| 20 | 19.50 | 24.75 | 28.00 | 30.50 | 30.25 | 27.75 | 24.00 | 31.25 | 29.75 | 28.00 | 24.00 | 22.75 |
| 21 | 19.25 | 28.25 | 27.00 | 31.75 | 29.50 | 28.50 | 27.00 | 30.50 | 28.00 | 28.25 | 25.50 | 22.50 |
| 22 | 19.75 | 28.25 | 27.00 | 29.75 | 23.00 | 27.00 | 28.00 | 31.00 | 29.00 | 27.75 | 26.00 | 22.50 |
| 23 | 22.00 | 27.25 | 28.50 | 27.50 | 21.75 | 27.00 | 27.25 | 31.75 | 25.50 | 28.25 | 25.00 | 22.00 |
| 24 | 22.50 | 23.75 | 28.25 | 26.50 | 25.50 | 27.25 | 26.00 | 32.50 | 27.00 | 27.75 | 24.00 | 24.00 |
| 25 | 23.50 | 23.25 | 29.00 | 25.00 | 26.00 | 29.25 | 23.00 | 28.25 | 25.25 | 28.25 | 26.25 | 24.25 |
| 26 | 23.50 | 23.25 | 29.25 | 27.00 | 27.50 | 23.25 | 25.50 | 30.50 | 24.50 | 29.25 | 26.75 | 24.50 |
| 27 | 24.75 | 20.25 | 26.00 | 27.00 | 27.50 | 25.25 | 26.00 | 29.50 | 26.75 | 29.25 | 25.75 | 20.50 |
| 28 | 24.50 | 24.00 | 28.00 | 31.00 | 29.25 | 25.75 | 26.75 | 31.75 | 27.50 | 31.00 | 25.75 | 19.75 |
| 29 | 22.50 | 25.75 | 30.00 | 31.25 | 30.50 | 26.75 | 28.50 | 32.25 | 28.25 | 29.75 | 25.00 | 20.75 |
| 30 | 22.25 | 27.50 | 29.50 | 33.50 | 29.75 | 27.50 | 29.75 |  | 28.50 | 28.25 | 24.50 | 24.00 |
| 31 | 22.00 | 26.00 |  | 31.50 |  | 27.75 | 30.75 |  | 29.25 |  | 20.75 |  |
