# Supplementary material for: A dynamic model for estimating adult female mortality from ovarian dissection data for the tsetse fly Glossina pallidipes Austen sampled in Zimbabwe
Source: PLoS Negl Trop Dis. 2017 Aug 30;11(8):e0005813. doi: 10.1371/journal.pntd.0005813 (PMC5576662; doi:10.1371/journal.pntd.0005813)
Supplement: S1 Additional Models — (DOCX) [file pntd.0005813.s005.docx]

**S1 Additional Models**

**Model with Age-Dependent Mortality**

As a sensitivity analysis, instead of estimating a single mature adult mortality, we allowed mature adult mortality to vary based on age according to the following [21]

$\mu\left( a \right)=k_{1}(k_{2}\exp\left( -k_{2}a \right)+k_{3}\exp\left( -k_{3}a \right))$

where $a$ is age of the fly in days, and $k_{1}$, $k_{2}$, and $k_{3}$ are parameters. Flies were assumed to have age equal to the midpoint age of their ovarian age. As shown in the table below, the parameters $\alpha$ and $\beta$ estimated by this model are comparable to $\alpha$ and $\beta$ estimated by model 3. To minimize the number of parameters to fit, $k_{3}$ was constrained to its literature value of 0.0112 per day [21], while $k_{1}$ and $k_{2}$ were allowed to vary. For comparability to models 1, 2, and 3, the mortalities for flies in ovarian ages 0 to 12 at temperatures below 25°C are given by the following: 0.064, 0.015, 0.017, 0.019, 0.021, 0.023, 0.025, 0.028, 0.031, 0.034, 0.038, 0.042, and 0.047, respectively.

**Table A**

| Quantity | Description | Value |
| --- | --- | --- |
| NLL/  AIC | Negative log of the probability of the model given the data/Akaike Information Criterion | 569.4/1152.8 |
| $S_{0}$ | Relative risk of capture for category 0 flies compared with category 2+ flies | 0.54  (0.51, 0.57) |
| $S_{1}$ | Relative risk of capture for category 1 flies compared with category 2+ flies. | 0.72  (0.68, 0.76) |
| $\alpha$ | Parameterizes increase in immature mortality with temperature. See Eq 4. [per degree C] | 0.86  (0.81, 0.92) |
| $\beta$ | Parameterizes increase in mature adult mortality with temperature. See Eq 3. [per degree C] | 0.0078  (0.0007, 0.083) |
| $d$ | Parameterizes increase in pupal mortality with increasing pupal density. See Eq 1. [per pupa per day] | 9.9x10^-7^  (1.5x10^-10^, 6.7x10^-3^) |
| $k_{1}$ | Parameterizes mature adult mortality. See equation above. [dimensionless] | 1.2  (1.1,1.3) |
| $k_{2}$ | Parameterizes mature adult mortality. See equation above. [per day] | 1.1  (0.98,1.2) |

**Model with Eight Days to First Ovulation**

As a sensitivity analysis, we constrained the time to first ovulation to be 8 days instead of 6, as assumed in our models presented in the main text. The model output below is for a model equivalent to model 3, except with an 8-day instead of 6-day time to first ovulation. The estimated mortality and dependence on temperature is consistent with model 3 in the main text.

**Table B**

| Quantity | Description | Value |
| --- | --- | --- |
| NLL/  AIC | Negative log of the probability of the model given the data/Akaike Information Criterion | 602.1/1216.1 |
| $S_{0}$ | Relative risk of capture for category 0 flies compared with category 2+ flies | 0.33  (0.31, 0.36) |
| $S_{1}$ | Relative risk of capture for category 1 flies compared with category 2+ flies. | 0.65  (0.62, 0.69) |
| $\alpha$ | Parameterizes increase in immature mortality with temperature. See Eq 4. [per degree C] | 0.87  (0.77, 0.99) |
| $\beta$ | Parameterizes increase in mature adult mortality with temperature. See Eq 3. [per degree C] | 0.012  (0.0034, 0.044) |
| $\mu_{a}$ | Per day mortality rate for mature adult female flies at temperatures less than 25°C. [per day]. | 0.027  (0.24, 0.32) |
| $d$ | Parameterizes increase in pupal mortality with increasing pupal density. See Eq 1. [per pupa per day] | 7.7x10^-6^  (2.8x10^-9^, 2.1x10^-2^) |
