# Supplementary material for: A dynamic model for estimating adult female mortality from ovarian dissection data for the tsetse fly Glossina pallidipes Austen sampled in Zimbabwe
Source: PLoS Negl Trop Dis. 2017 Aug 30;11(8):e0005813. doi: 10.1371/journal.pntd.0005813 (PMC5576662; doi:10.1371/journal.pntd.0005813)
Supplement: S1 Equations — (DOCX) [file pntd.0005813.s006.docx]

**S1 Equations**

$$\frac{dP}{dt}= - \mu_{p}(T,P)P+\frac{1}{9} \frac{1}{2} \sum_{i=1}^{7} C_{i}-h\left( T \right)P$$

$$\frac{dC_{1}}{dt}= - \mu_{a}(T)C_{1}-\frac{1}{6} C_{1}+h(T)P$$

*For* $i\geq1$:

$$\frac{dC_{i}}{dt}= - \mu_{a}(T)C_{i}-\frac{1}{9} C_{i}+\frac{1}{9} {(1-\delta_{1i})C}_{i-1}+\frac{1}{6} \delta_{1i}C_{0}+\delta_{4i}\frac{1}{9} C_{7}$$

where $P$ is the number of pupa, $C_{i}$ is the number of adults in ovarian category *i* (categories 0 to 7)*, T* is the temperature, $h(T)$ is the temperature-dependent pupal emergence rate given in equation 2, and $\delta_{ij}$ is the Kronneker delta function ($\delta_{ij}$ is equal to 1 if *i=j* and 0 otherwise). All other parameters are given in Table 1.
